# Perception versus reality: A National Cohort Analysis of the surgery‐first approach for resectable pancreatic cancer

**DOI:** 10.1002/cam4.4144

**Published:** 2021-07-21

**Authors:** John R. Bergquist, Cornelius A. Thiels, Christopher R. Shubert, Tommy Ivanics, Elizabeth B. Habermann, Santhi S. Vege, Travis E. Grotz, Sean P. Cleary, Rory L. Smoot, Michael L. Kendrick, David M. Nagorney, Mark J. Truty

**Affiliations:** ^1^ Department of Surgery Division of Surgical Oncology Stanford University Stanford, Palo Alto California USA; ^2^ Division of Hepatobiliary and Pancreas Surgery Mayo Clinic Rochester Rochester Minnesota USA; ^3^ Department of Surgery Division of Surgical Oncology Memorial Sloan‐Kettering Cancer Center New York New York USA; ^4^ Department of Surgery Division of Hepatopancreatobiliary Surgery Johns Hopkins University Baltimore Maryland USA; ^5^ Department of Surgery Henry Ford Medical Center Detroit Michigan USA; ^6^ Mayo Clinic Robert D. and Patricia E. Kern Center for the Science of Health Care Delivery Rochester Minnesota USA; ^7^ Mayo Clinic Rochester, Department of Medicine, Division of Gastroenterology Pancreatology Section Rochester Minnesota USA

**Keywords:** adjuvant therapy, neoadjuvant, pancreatic cancer, pancreaticoduodenectomy, resectability

## Abstract

**Introduction:**

Although surgical resection is necessary, it is not sufficient for long‐term survival in pancreatic ductal adenocarcinoma (PDAC). We sought to evaluate survival after up‐front surgery (UFS) in anatomically resectable PDAC in the context of three critical factors: (A) margin status; (B) CA19‐9; and (C) receipt of adjuvant chemotherapy.

**Methods:**

The National Cancer Data Base (2010–2015) was reviewed for clinically resectable (stage 0/I/II) PDAC patients. Surgical margins, pre‐operative CA19‐9, and receipt of adjuvant chemotherapy were evaluated. Patient overall survival was stratified based on these factors and their respective combinations. Outcomes after UFS were compared to equivalently staged patients after neoadjuvant chemotherapy on an intention‐to‐treat (ITT) basis.

**Results:**

Twelve thousand and eighty‐nine patients were included (*n *= 9197 UFS, *n* = 2892 ITT neoadjuvant). In the UFS cohort, only 20.4% had all three factors (median OS = 31.2 months). Nearly 1/3rd (32.7%) of UFS patients had none or only one factor with concomitant worst survival (median OS = 14.7 months). Survival after UFS decreased with each failing factor (two factors: 23 months, one factor: 15.5 months, no factors: 7.9 months) and this persisted after adjustment. Overall survival was superior in the ITT‐neoadjuvant cohort (27.9 vs. 22 months) to UFS.

**Conclusion:**

Despite the perceived benefit of UFS, only 1‐in‐5 UFS patients actually realize maximal survival when known factors highly associated with outcomes are assessed. Patients are proportionally more likely to do worst, rather than best after UFS treatment. Similarly staged patients undergoing ITT‐neoadjuvant therapy achieve survival superior to the majority of UFS patients. Patients and providers should be aware of the false perception of ‘optimal’ survival benefit with UFS in anatomically resectable PDAC.

## Graphical abstract

Survival of resectable pancreatic cancer patients treated with surgery and stratified by margin status, CA19‐9 levels, and chemotherapy receipt shows survival is highly predicted by these factors and is proportionally not ideal in the majority of undergoing an upfront resection strategy. Neoadjuvant chemotherapy offers significant benefit to upfront surgery in the context of acheiving optimal survival in the context of these factors.

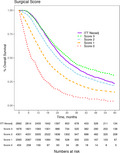

## 1 INTRODUCTION

Surgical resection is necessary for long‐term survival in pancreatic ductal adenocarcinoma (PDAC) and is therefore perceived as the optimal initial treatment strategy in the 15%–20% of patients presenting with anatomically ‘resectable’ tumors.^1^ However, despite curative‐intent, surgery is not sufficient for durable long‐term survival as the majority of patients undergoing resection develop postoperative recurrence.^2^ The concept of ‘borderline’ resectability was introduced to identify those patients that may benefit from neoadjuvant therapy prior to resection due to increased risk of positive margins with upfront resection.^3^ Although a neoadjuvant strategy is increasingly utilized in anatomically borderline PDAC, there is no consensus on the benefit of this approach in anatomically resectable tumors, despite increased interest in such an approach.^4^, ^5^, ^6^ What is known based on previously established data, is that specific factors profoundly influence post‐operative survival in those patients undergoing upfront surgery (UFS): margin status, CA19‐9 levels, and receipt of adjuvant chemotherapy. Despite improvements in cross‐sectional radiologic imaging, at least 20%–30% of anatomically resectable patients will have a positive margin (R1) resection with consequent approximately 50% decrease in median survival.^7^, ^8^ The importance of elevated CA19‐9 levels as a surrogate of occult metastatic disease and early postoperative recurrence is now recognized.^9^, ^10^, ^11^, ^12^ Finally, multiple trials have demonstrated the survival benefit of adjuvant systemic chemotherapy in resectable pancreatic cancer, suggesting the likelihood of occult residual disease in the majority of patients undergoing curative upfront resection. However, many patients do not ultimately begin or complete this recommended therapy.^13^, ^14^, ^15^, ^16^, ^17^, ^18^, ^19^ Although there have been several large database studies comparing upfront resection (UFS) with neoadjuvant therapy in anatomically resectable PDAC, none have specifically evaluated outcomes in the context of these important factors that profoundly and independently impact survival.^4^, ^5^, ^20^, ^21^, ^22^

In the era of UFS for PDAC, improvements in overall survival have plateaued. Thus, although surgery is known to be necessary for long‐term survival in patients with pancreatic cancer, it is not sufficient to guarantee durable survival due to the influence of the factors outlined above. Due to the extensive resources required to conduct randomized trials in oncology, the challenges presented by the inability to blind patients to a neoadjuvant versus upfront‐surgery treatment strategy, and the time which would be required to measure survival outcomes, such studies are difficult to appropriately design and accrue practically. Consequently, the utilization of observational data can facilitate the current critical evaluation of the utility of UFS in localized PDAC.

Therefore, the present study is designed to assess the combinatorial frequency of the absence or presence of the above‐mentioned survival factors in patients with localized, anatomically resectable pancreatic cancer undergoing a surgery‐first approach. Such an analysis to our knowledge has not been performed before. In contrast to prior work, this study does not merely intend to directly compare UFS against neoadjuvant, but rather seeks to add context by the determination of the ‘proportion’ of patients that are able to achieve optimal outcomes with upfront resection—a critical measure for population oncology. The aim was to assess the individual and combined influence of these factors on postoperative survival. For contrast, outcomes were compared to similarly staged patients undergoing neoadjuvant systemic chemotherapy on an intent‐to‐treat basis, including patients who did not proceed to resection.

## 2 PATIENTS AND METHODS

This study is a retrospective cohort analysis of the National Cancer Data Base (NCDB) participant user file (PUF) of patients undergoing treatment for localized (AJCC Stage 0/I/II) pancreatic adenocarcinoma from 2010 to 2015. The Mayo Clinic Institutional Review Board has deemed analysis of the NCDB PUF exempt from review. The NCDB contains over 30 million individual cancer cases collected by more than 1500 Commission on Cancer (CoC) approved facilities across the United States (US) and reportedly captures over 70% of newly diagnosed cases of cancer in the US.^23^

**FIGURE 1:**
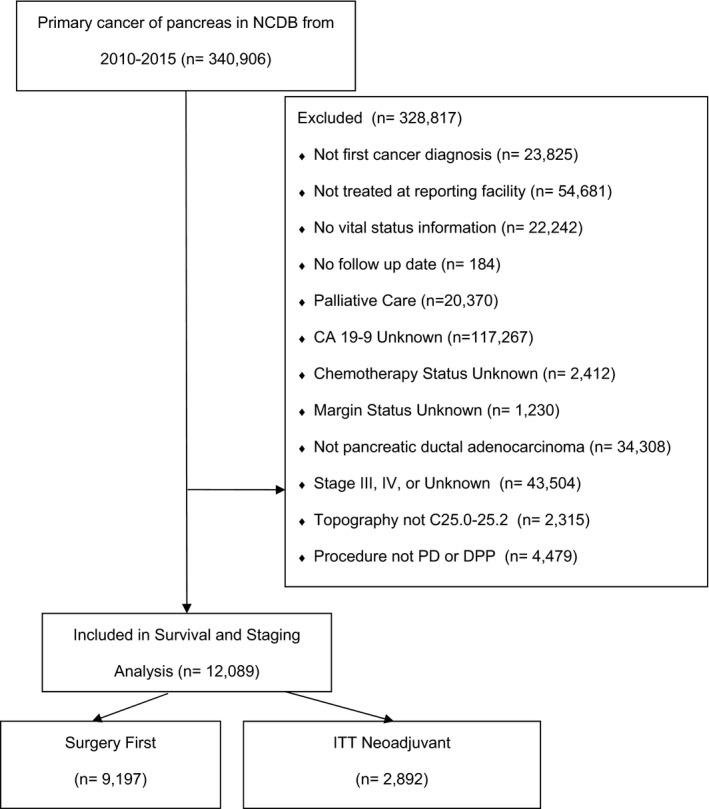
STROBE diagram of cohorts included and excluded

Patients with PDAC were identified using International Classification of Diseases for Oncology, third edition (ICD‐O‐3) topography (C25.0–C25.9) and histology (8140–8145, 8211, 8230, 8260–8263, 8290, 8310, 8480–8481, 8500–8508, 8521–8523, 8570–8576) codes. Included patients were diagnosed and treated at the reporting facility. Patients diagnosed with multi‐site cancer and those missing pathologic or follow‐up data were excluded. Summary staging was assessed using the sixth or seventh edition AJCC staging manual according to the year of the case. Curative intent surgery included surgery of primary site codes 21–89 which includes pancreaticoduodenectomy, distal partial pancreatectomy, total pancreatectomy, and pancreatectomy NOS. Patients with surgical codes 0 (no surgery), 90 (surgery, NOS), and 99 (unknown) were excluded. Patient data were assessed for: (A) negative (R0) margins; (B) normal pre‐op CA19‐9 levels (<37 U/ml), evaluated according to previously published methods^12^; and (C) receipt of any adjuvant chemotherapy (single or multi‐agent). Patients undergoing UFS were scored based on the absence (score = 0) or presence (score = 1) of any combination of these factors (total score = 0–3) and overall survival (OS) was stratified by various factorial combinations. We then compared these outcomes with similarly staged patients undergoing neoadjuvant chemotherapy on an intention to treat (ITT) basis, including those treated with neoadjuvant therapy but who did not subsequently undergo curative‐intent surgery. Missing data were handled with case exclusion or indicator variables. A STROBE‐compliant diagram showing all patients included and excluded is provided in Figure 1.

### 2.1 Statistical analysis

Normally distributed continuous data were expressed as mean and standard deviation and interrogated for equivalence with the two‐tailed Student’s *t*‐test. Non‐normally distributed continuous data were expressed as median (inter‐quartile range) interrogated for equivalence with the Mann–Whitney U test. Pearson’s chi‐squared tests were used to interrogate uniformly distributed categorical variables and Fisher’s exact test was used for categorical variables with non‐uniform distribution. Unadjusted survival analysis was performed using the method of Kaplan and Meier with survival defined as the time in months from date of surgery to death with THE censorship of patients alive at last follow‐up. Unadjusted survival estimates were compared by means of the log‐rank test. The NCDB does not provide data on progression or recurrence, therefore survival is reported overall. To estimate the impact of procedure type and adjuvant therapy receipt on survival, we constructed a multivariable Cox proportional hazards model adjusted for age, gender, race, Charlson‐Deyo comorbidity score, grade, presence of lymphovascular invasion, margin status, receipt of radiation, receipt of chemotherapy, type of surgery, and type of facility. A significance level of 0.05 was used for all comparisons. Statistical analysis was performed with R version 3.5.2 (‘Eggshell Igloo’—R Foundation for Statistical Computing, www.r‐project.org).

## 3 RESULTS

Twelve thousand and eighty‐nine total patients were included, of which 9197 (76%) underwent UFS and 2892 (24%) received neoadjuvant chemotherapy with a plan to proceed with curative surgery (ITT). Descriptive characteristics and details for each cohort are shown in Table 1. UFS patients were slightly older (median age at diagnosis 66 vs. 63), less often caucasian (84.4% vs. 87.2%), had higher comorbidity (64.9% vs. 67.4% Charlson score 0), and were less often treated in the academic setting (56.8% vs. 65.5%, all *p *< 0.001) compared to the ITT neoadjuvant cohort. There was no difference in gender between the groups. Differences in tumor location between groups were small, but UFS patients were less likely to have their tumor in the head of the pancreas.

**TABLE 1:** Cohort demographics, pathologic characteristics, and outcomes

	Surgery‐first (UFS)	Neoadjuvant (ITT)	*p*
	*n* = 9197	*n* = 2892	
Median age [IQR]	66.0 [59.0, 74.0]	63.0 [56.0, 69.0]	<0.001
Female sex	48.8%	47.6%	0.286
Race			0.002
Caucasian	84.4%	87.2%	
African American	10.8%	9.2%	
Other	4.8%	3.6%	
Charlson Deyo score			0.028
0	64.9%	67.4%	
1	29.0%	28.0%	
2+	6.1%	4.6%	
Facility type			<0.001
Community	3.3%	2.8%	
Comprehensive community	27.9%	21.7%	
Academic/research	56.8%	65.5%	
Integrated network	12.0%	9.9%	
Year of diagnosis			<0.001
2010	18.7	13.6	
2011	19.6	16.7	
2012	20.5	19.3	
2013	21.1	22.3	
2014	20.0	28.1	
Clinical stage (%)			<0.001
Stage I	34.4%	23.6%	
Stage II	39.1%	55.4%	
Missing	26.5%	21.0%	
Final path stage (%)			<0.001
Stage I	8.8%	20.3%	
Stage IIA	21.5%	32.2%	
Stage IIB	69.7%	47.5%	
Location			<0.001
Head	75.0%	78.4%	
Body/tail	15.5%	11.3%	
Other/NOS	9.5%	10.3%	
Median tumor size (cm) [IQR]	3.2 [2.5, 4.1]	3.2 [2.5, 4.1]	0.762
CA 19‐9 elevated above normal	69.2%	62.8%	<0.001
Tumor size category			<0.001
<2 cm	10.3%	9.3%	
>2 cm	88.5%	87.3%	
Missing	1.2%	3.5%	
cN status			
cN0	62.6%	63.9%	
cN1	21.0%	29.5%	
Missing or unstaged	16.4%	6.6%	
pN1 status (% of available)	70.7%	47.6%	<0.001
High grade (% of available)	37.3%	32.2%	<0.001
Lymphovascular invasion (% of available)	53.4%	34.7%	<0.001
Positive margin (% of available)	23.7%	16.7%	<0.001
Any radiation	31.7%	62.5%	<0.001
Adjuvant chemotherapy	69.6%	33.8%	<0.001
30‐day readmission	7.9%	5.9%	<0.001
90‐day mortality	5.3%	4.5%	<0.001

**FIGURE 2:**
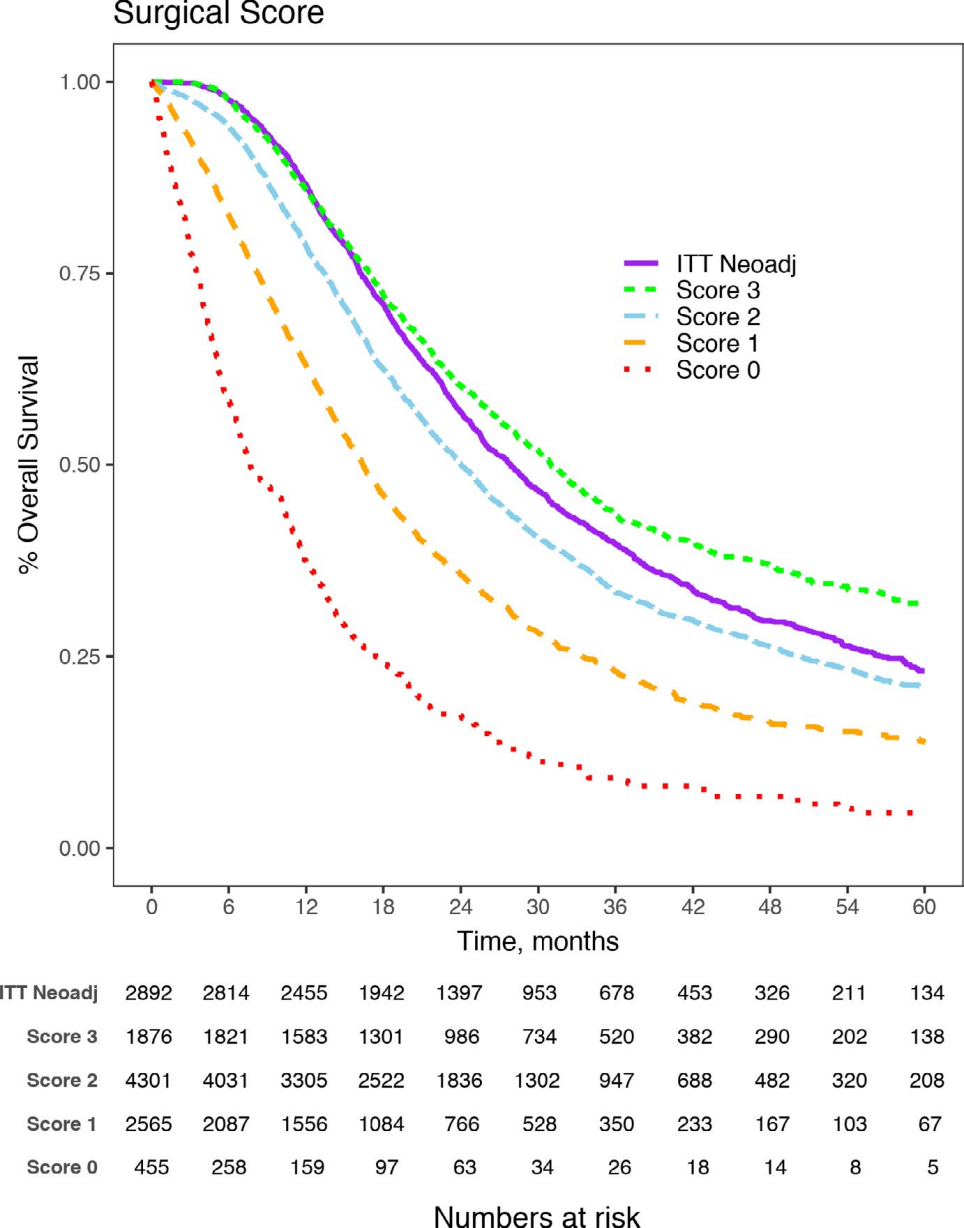
Unadjusted survival analysis

**FIGURE 3:**
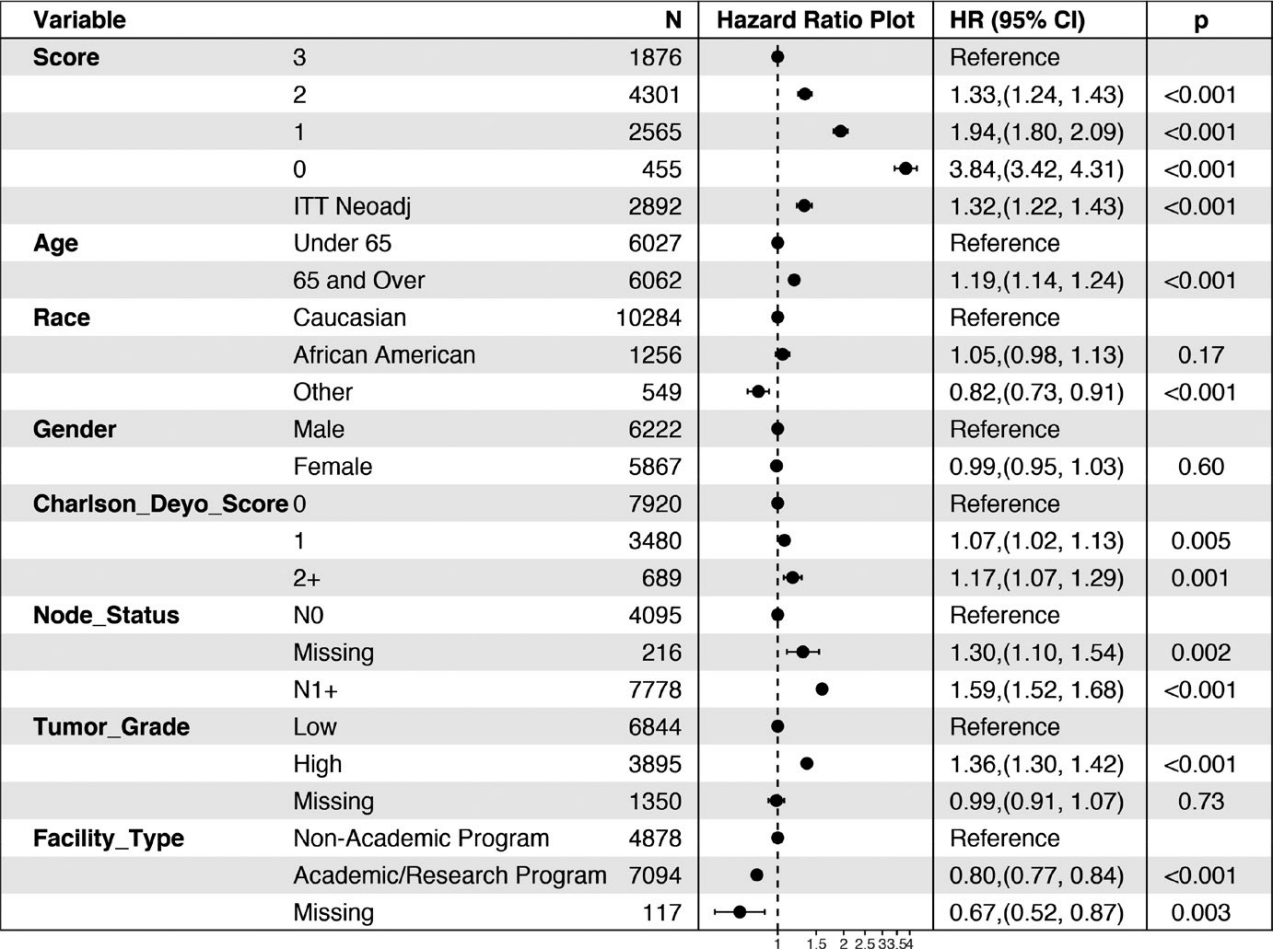
Adjusted proportional hazards survival model

Among UFS patients, 76.3% had negative margin resections, 31.8% had non‐elevated CA19‐9 levels, and 69.6% received adjuvant chemotherapy. Only 20.4% of UFS patients had all three of these survival factors that resulted in the best median OS of 31.2 months (Table 2). In contrast, nearly 1/3rd of patients (32.7%) treated with UFS had either one or none of these survival factors, and this was associated with the worst survival (median OS = 14.7 months). Unadjusted median survival decreased with the absence of each survival factor (23.4 months for two factors, 46.8% of patients; 15.5 months for one factor, 27.9% of patients; and 7.9 months for no factors, 4.9% of patients), and this survival varied by specific factorial combinations as outlined in Table 2. Figure 2 graphically shows the unadjusted overall survival of the UFS patients stratified by their total factor score. The decrease in survival with the absence of each factor persisted even after adjustment for other clinically significant variables (age, gender, race, comorbidity score, node status, tumor grade, type of facility—Figure 3).

**TABLE 2:** Mortality hazard and unadjusted median overall survival based on the score/treatment category

Treatment strategy	Score category	% (*N*) of Pts.			Unadjusted median overall survival (months)		
Surgery‐first	ABC score = 3	20.4% (1876)			31.0		
	AC only, score = 2	32.4% (2977)	46.8% (4301)	79.6% (7321)	23.9	23.4	19.6
	AB only, score = 2	8.4% (772)			23.3		
	BC only, score = 2	6.0% (552)			21.3		
	C only, score = 1	10.9% (999)	32.8% (3020)		17.5	14.7	
	A only, score = 1	14.7% (1349)			15.2		
	B only, score = 1	2.4% (217)			10.4		
	None score = 0	4.9% (455)			7.9		
ITT neoadjuvant	—	100% (2892)			27.9		

Note:

A = Negative resection margin; B = Normal CA 19‐9; C = Receipt of adjuvant chemotherapy.

Over the course of the study period, the proportion of patients undergoing ITT neoadjuvant chemotherapy significantly increased from 18.6% in 2010 to 30.7% in 2014, and these patients were compared to UFS patients on clinical stage (0/I/II) at presentation (Table 1). Compared to UFS patients, the ITT neoadjuvant cohort was slightly less likely to have elevated CA 19‐9 (62.8% vs. 69.2%). ITT neoadjuvant patients were more likely to have clinical stage II disease (55.4% vs. 39.1%) and higher rates of clinically node‐positive disease (29.5% vs. 21.0%) at diagnosis. In spite of this, final pathologic markers for ITT neoadjuvant patients undergoing resection skewed favorably compared to the UFS cohort with ITT patients much less likely to have final pathologic stage II disease after resection (79.7% vs. 91.2%), less positive nodes (47.6% vs. 70.7%), lower lymphovascular invasion (34.7% vs. 53.4%), and lower rate of positive surgical margins (16.7% vs. 23.7%) suggesting treatment response to neoadjuvant therapy. Unplanned readmission within 30 days was more common (7.9% vs. 5.9%) and the mortality rate at 90 days after surgery was higher (5.3% vs. 4.5%) in the UFS cohort compared to those in the ITT neoadjuvant cohort. The overall survival for all patients in this study was 23.5 months and was superior in the ITT neoadjuvant cohort (27.9 months) versus UFS (22.0 months). Median OS in the ITT neoadjuvant cohort was better than the survival in 80% of UFS patients. All comparisons reported above met statistical significance criteria.

## 4 DISCUSSION

Despite the perceived benefit of a surgery‐first strategy in patients with localized, resectable PDAC,^24^ only 1‐in‐5 (20%) patients actually achieved maximum survival in the context of these three established predictive factors (margin status, pre‐op CA19‐9 levels, and receipt of adjuvant chemotherapy). Survival decreased with each failing factor and a high proportion of patients (34%) were identified with only one or no factors with the resultant worst survival overall. The analysis confirms that UFS, although perceived as maximally beneficial, is in fact highly dependent on these specific factors, which apart from preoperative CA19‐9 levels, cannot be known or guaranteed prior to resection. Therefore, despite the dominant perception of the benefit of UFS in anatomically resectable pancreatic cancer, the reality is that the majority of patients have a higher likelihood of actually doing the worst, rather than the best in the context of these factors with upfront resection. This is a probability that most surgeons and patients likely do not consider or realize, and should give pause to such a practice. These data also further highlight the potential benefits of neoadjuvant treatment sequencing, even in patients with surgically resectable PDAC.^25^ These findings have an impact on treatment standards and guidelines.

In this study, 23.7% of patients had positive margins on final pathologic analysis, consistent with rates from previous adjuvant trials in resectable PDAC. In fact, this rate of margin positivity was lower than the ESPAC‐III data (35%), but higher than CONKO (17%) and ESPAC‐1 (18%).^15^, ^26^, ^27^ CA19‐9 was elevated in over 60% of patients in this cohort, and this is also highly consistent with rates reported in prior studies.^8^, ^28^, ^29^ Prior work has shown that any elevation of CA19‐9 above normal is associated with detrimental stage‐matched survival outcomes, and that the only treatment sequence which ameliorates the decreased survival associated with CA19‐9 elevation is neoadjuvant systemic chemotherapy.^12^ Despite its known negative influence as well as reports from multiple centers on specific optimal CA19‐9 cutoff levels, this important and predictive survival factor continues to be under‐utilized in otherwise resectable PDAC patients—demonstrated by prior data revealing that only approximately 25% of patients in national datasets even have pre‐operative CA19‐9 measured.^12^ In the present study, 30.4% of patients did not receive adjuvant chemotherapy, and this is similar to the proportion observed in other studies of adjuvant therapy in PDAC.^29^, ^30^, ^31^ Although prior analysis using the NCDB dataset compared survival outcomes of surgery‐first versus neoadjuvant treatment in resectable PDAC,^5^, ^20^, ^21^, ^22^ this is the first study to specifically compare outcomes of each treatment sequence in the context of these critical and previously established survival factors in resectable patients. Therefore, this analysis provides much‐needed clinical insight into the influence of these survival factors, two of which (margin status and adjuvant therapy receipt) cannot be reliably predicted with the surgery‐first approach.

Despite improvements in modern radiologic imaging, final pathologic margin status cannot be reliably predicted *a priori*. This is evident in numerous adjuvant chemotherapy trials for resectable PDAC that revealed positive margins in 17%–35% of patients despite strict radiologic enrollment protocols for anatomically resectable tumors.^15^, ^26^, ^27^ Although modern imaging can predict major vascular involvement, it cannot determine extra‐pancreatic extension and microscopic tumor infiltration, pathologic hallmarks of PDAC. Despite the improved survival outcomes with adjuvant chemotherapy, these same trials have demonstrated that a significant proportion of patients do not ever receive or complete systemic therapy postoperatively for a variety of reasons (38% in CONKO‐001, 50% in ESPAC‐I, and 43% in ESPAC III).^15^, ^26^, ^27^ Although CA19‐9 levels can be ascertained at diagnosis and prior to treatment, and its impact on survival is well known, unfortunately to date these findings have not led to significantly altered treatment guidelines.^9^

Aside from the clear survival benefit conveyed by the pursuit of ITT neoadjuvant therapy at the time of diagnosis, the data presented here also suggest that ITT neoadjuvant therapy is associated with improvement in other predictors of postoperative survival which may contribute to the overall impact. In spite of a higher rate of clinically positive nodes, the ITT neoadjuvant cohort had a lower rate of node positivity on final pathology, suggesting the treatment effect. Node status is a clearly established predictor of worse survival—as evidenced by its presence in the AJCC staging system. Additionally, the rates of pathologic high grade and lymphovascular invasion were lower in the ITT neoadjuvant group, which may suggest treatment effect and the concomitant impact on long‐term survival, or could be due to selection. Finally, it is noteworthy that patients treated in ITT‐neoadjuvant fashion in this study were more commonly clinical stage II and had longer survival in spite of this, suggesting an actual inversion of the staging system associated with neoadjuvant therapy. This suggests that for patients treated with a neoadjuvant approach, modifications to the staging system may be warranted.^32^, ^33^

It is noteworthy that the dropout rate from ITT neoadjuvant therapy in this study was very low and may not accurately represent the true dropout rate given the limitations associated with such national datasets. There is a paucity of existing literature on dropout frequency after neoadjuvant therapy administration in resectable pancreatic cancer. The recent SWOG‐S1505 trial abstract presented at ASCO 2020 had approximately 6% dropout after neoadjuvant chemotherapy initiation but prior to resection.^34^ The publication of more data on the rate of progression in clinically resectable pancreatic cancer patients treated with ITT neoadjuvant therapy is needed to understand the risks associated with this approach.

The best method for evaluating therapeutic efficacy in oncologic care has traditionally been the randomized controlled trial, which is viewed as the ‘gold standard’ of clinical evidence. However, in recent years, there is a growing recognition that trials can hinder our understanding of oncologic disease due to their cost, time required to execute, and the challenge of blinding patients to interventions such as neoadjuvant chemotherapy versus UFS as well as creating an optimal design to avoid confounding.^35^, ^36^, ^37^ Although randomized trials of UFS versus neoadjuvant systemic chemotherapy are being conducted, they are not blinded and their generalizability may be limited. In contrast, the utilization of observational ‘big data’ sources such as the NCDB can facilitate understanding of how treatment decisions can be optimized based on true ‘real world’ evidence. Although there is significant bias and confounding in these types of studies, there is growing recognition that well‐designed studies utilizing observational data can provide strong insight into clinical best practices in oncology.^38^ Real‐world observational data such as these are useful for understanding actual practice patterns and realized outcomes and should be utilized to inform the design and enrollment criteria for future randomized study protocols.^39^ In this way, retrospective analysis of ‘big data’ such as these can help advance the ‘gold standard’ in pancreatic cancer care.

Furthermore, the use of randomization to study oncologic therapy has major limitations which are often overlooked: fundamental flaws in design, limited power, and problems with randomization execution. For example, although the recently presented SWOG‐S1505 data demonstrate the feasibility of producing high‐quality randomized data in this field, the fact that the study took 5 years to produce and did not demonstrate a clear ‘winner’ despite the ‘pick the winner’ design provides evidence of the limitations of studying cancer therapeutics using the methodology of randomization.^34^ Additionally, the ESPAC‐5F results failed to demonstrate a clear clinical difference, showing that even with national scope in a country with 70 million inhabitants over 4 years, there is insufficient accrual to execute a randomized study with four therapy arms and show a clinically meaningful difference.^40^ If randomization is the only method used to study pancreatic cancer, there will be many more years and patients lost before a high‐quality answer to the question of whether UFS or neoadjuvant chemotherapy is a better treatment for patients with resectable pancreatic cancer is available. In the meantime, the data presented here can provide valuable perspective for clinical decision making both for the surgeon and the patient. The question posed should not be ‘is UFS better than neoadjuvant chemotherapy in resectable PDAC’ in general, but rather what is the quantitative proportional benefit and odds for patients based on various known and established survival factors with an upfront surgical approach. Our data would suggest that survival is realistically far worse and durable survival is less likely than perceived in the context of this survival‐factor‐based analysis.

### 4.1 Limitations

This study is limited by its retrospective and non‐randomized nature. We have attempted to utilize careful cohort selection and outlier exclusion to reduce the effects of selection bias and provide transparency into the sources of bias present in our analysis. NCDB does not provide data on the completion of non‐surgical therapy therefore we cannot ascertain the completeness of chemotherapy and/or radiation treatment. Clinical staging data in the NCDB—as with all secondary data sources—may be incomplete or inaccurate, thus impacting the results of this analysis. Surgery‐specific complication data such as post‐operative pancreatic fistula or delayed gastric emptying are not available although these are some of the main contributing factors to non‐receipt of adjuvant therapy in this population. Only one CA 19‐9 level is provided in the NCDB, with limited granularity as we have previously described.^12^ However, the date when it was measured is not included, nor is this field a required field, and it is missing in many cases. Therefore, there is no way to utilize multiple CA 19‐9 levels to measure for example treatment response. Data on the exact type of systemic chemotherapy and quantification of therapy administered are lacking in the NCDB, precluding analysis of the extent of systemic therapy administered. Completeness of the pathologic analysis is not possible to assess based on the data available in the NCDB. Finally, there is no data in NCDB on the utilization of salvage systemic therapy in response to recurrence or progression.

## 5 CONCLUSION

Complete surgical extirpation followed by adjuvant systemic chemotherapy remains the ‘gold standard’ therapy for resectable pancreatic cancer. In spite of the perception that this surgery‐first strategy in ‘resectable’ PDAC is maximally beneficial, only 1‐in‐5 patients are able to achieve maximal oncologic benefit when assessed in the context of three known survival factors: negative margins, normal CA19‐9, and receipt of adjuvant chemotherapy. Survival decreases with each failing factor, two of which cannot be predicted prior to surgery (margin status, adjuvant therapy). When assessed by these factors, patients with resectable tumors treated with a surgery‐first approach are more likely to have the worst rather than the best survival outcomes. Similarly staged patients undergoing ITT‐neoadjuvant therapy can achieve survival outcomes superior to the majority of patients treated with the surgery‐first approach. Patients should be counseled regarding their actual probability of achieving maximal survival benefit rather than the perceived probability when discussing options for therapeutic sequencing. Further investigation is needed to critically re‐assess the perceived benefit compared to actual outcomes in a surgery‐first treatment paradigm for resectable PDAC with less focus on resection itself as an arbitrary metric of oncologic success and more attention paid to specific and established predictive factors of surgery outcomes.

## 6 ETHICS STATEMENT

The Mayo Clinic Institutional Review Board has deemed analysis of the NCDB PUF exempt from review, and a blinded statement to this effect is in the methods section of the manuscript.

## CONFLICT OF INTEREST

All authors disclose no conflict of interest related to this work.

## AUTHOR CONTRIBUTIONS

Bergquist participated in the conception, design, acquisition of data, analysis, and interpretation. Thiels, Shubert, Ivanics participated in the design, analysis, and interpretation. Habermann, Vege, Grotz, Cleary, Smoot, Kendrick, and Nagorney participated in the interpretation of the data. Truty participated in the conception, design, and interpretation. All authors participated in drafting the article and gave final approval.

## Data Availability

The data that support the findings of this study are available from the Commission on Cancer. Restrictions apply to the availability of these data, which were used under license for this study. Data are available by direct request to the Commission on Cancer/NCDB.

## References

[1] Kleeff J, Korc M, Apte M, et al. Pancreatic cancer. Nat Rev Dis Prim. 2016;2:16022. 10.1038/nrdp.2016.22. 27158978

[2] Nishio K, Kimura K, Amano R, et al. Preoperative predictors for early recurrence of resectable pancreatic cancer. World J Surg Oncol. 2017;15(1):16. 10.1186/s12957-016-1078-z. 28069033 PMC5223494

[3] Katz MHG, Pisters PWT, Evans DB, et al. Borderline resectable pancreatic cancer: the importance of this emerging stage of disease. J Am Coll Surg. 2008;206(5):833‐838. 10.1016/j.jamcollsurg.2007.12.020. 18471707 PMC5901743

[4] Lee AJ, Simoneau E, Chiang Y‐J, et al. Is early‐stage pancreatic adenocarcinoma truly early: stage migration on final pathology with surgery‐first versus neoadjuvant therapy sequencing. HPB. 2019;21(9):1203‐1210. 10.1016/j.hpb.2019.01.011. 30799277

[5] Mokdad AA, Minter RM, Zhu H, et al. Neoadjuvant therapy followed by resection versus upfront resection for resectable pancreatic cancer: a propensity score matched analysis. J Clin Oncol. 2017;35(5):515‐522. 10.1200/JCO.2016.68.5081. 27621388

[6] Bergquist JR, Shubert CR, Storlie CB, Habermann EB, Truty MJ. Patient selection for neoadjuvant therapy in early‐stage pancreatic cancer. J Clin Oncol. 2017;35(14):1622‐1623. 10.1200/JCO.2016.71.2315. 28135141

[7] Tummers WS, Groen JV, Sibinga Mulder BG, et al. Impact of resection margin status on recurrence and survival in pancreatic cancer surgery. Br J Surg. 2019;106(8):1055‐1065. 10.1002/bjs.11115. 30883699 PMC6617755

[8] Ghaneh P, Kleeff J, Halloran CM, et al. The impact of positive resection margins on survival and recurrence following resection and adjuvant chemotherapy for pancreatic ductal adenocarcinoma. Ann Surg. 2019;269(3):520‐529. 10.1097/SLA.0000000000002557. 29068800

[9] Hartwig W, Strobel O, Hinz U, et al. CA19‐9 in potentially resectable pancreatic cancer: perspective to adjust surgical and perioperative therapy. Ann Surg Oncol. 2013;20:2188‐2196. 10.1245/s10434-012-2809-1. 23247983

[10] Kondo N, Murakami Y, Uemura K, et al. Prognostic impact of perioperative serum CA 19‐9 levels in patients with resectable pancreatic cancer. Ann Surg Oncol. 2010;17(9):2321‐2329. 10.1245/s10434-010-1033-0. 20336387

[11] Barton JG, Bois JP, Sarr MG, et al. Predictive and prognostic value of CA 19–9 in resected pancreatic adenocarcinoma. J Gastrointest Surg. 2009;13:2050‐2058. 10.1007/s11605-009-0849-z. 19756875

[12] Bergquist JR, Puig CA, Shubert CR, et al. Carbohydrate antigen 19–9 elevation in anatomically resectable, early‐stage pancreatic cancer is independently associated with decreased overall survival and an indication for neoadjuvant therapy: a national cancer database study. J Am Coll Surg. 2016;223(1):52‐65. 10.1016/j.jamcollsurg.2016.02.009. 27049786

[13] Sohn TA, Yeo CJ, Cameron JL, et al. Resected adenocarcinoma of the pancreas‐616 patients: results, outcomes, and prognostic indicators. J Gastrointest Surg. 2000;4(6):567‐579. 11307091 10.1016/s1091-255x(00)80105-5

[14] Murakami Y, Uemura K, Sudo T, et al. Early initiation of adjuvant chemotherapy improves survival of patients with pancreatic carcinoma after surgical resection. Cancer Chemother Pharmacol. 2013;71(2):419‐429. 10.1007/s00280-012-2029-1. 23178955

[15] Oettle H, Neuhaus P, Hochhaus A, et al. Adjuvant chemotherapy with gemcitabine and long‐term outcomes among patients with resected pancreatic cancer: the CONKO‐001 randomized trial. JAMA. 2013;310(14):1473‐1481. 10.1001/jama.2013.279201. 24104372

[16] Von Hoff DD, Ervin T, Arena FP, et al. Increased survival in pancreatic cancer with nab‐paclitaxel plus gemcitabine. N Engl J Med. 2013;369(18):1691‐1703. 10.1056/NEJMoa1304369. 24131140 PMC4631139

[17] Conroy T, Desseigne F, Ychou M, et al. FOLFIRINOX versus gemcitabine for metastatic pancreatic cancer. N Engl J Med. 2011;364(19):1817‐1825. 10.1056/NEJMoa1011923. 21561347

[18] Dhir M, Malhotra GK, Sohal DPS, et al. Neoadjuvant treatment of pancreatic adenocarcinoma: a systematic review and meta‐analysis of 5520 patients. World J Surg Oncol. 2017;15(1):183. 10.1186/s12957-017-1240-2. 29017581 PMC5634869

[19] Liao W‐C, Chien K‐L, Lin Y‐L, et al. Adjuvant treatments for resected pancreatic adenocarcinoma: a systematic review and network meta‐analysis. Lancet Oncol. 2013;14(11):1095‐1103. 10.1016/S1470-2045(13)70388-7. 24035532

[20] Shubert CR, Bergquist JR, Groeschl RT, et al. Overall survival is increased among stage III pancreatic adenocarcinoma patients receiving neoadjuvant chemotherapy compared to surgery first and adjuvant chemotherapy: an intention to treat analysis of the National Cancer Database. Surgery. 2016;160(4):1080‐1096. 10.1016/j.surg.2016.06.010. 27522556

[21] Mirkin KA, Hollenbeak CS, Wong J. Survival impact of neoadjuvant therapy in resected pancreatic cancer: a prospective cohort study involving 18,332 patients from the National Cancer Data Base. Int J Surg. 2016;34:96‐102. 10.1016/j.ijsu.2016.08.523. 27573691

[22] Shridhar R, Takahashi C, Huston J, Meredith KL. Neoadjuvant therapy and pancreatic cancer: a national cancer database analysis. J Gastrointest Oncol. 2019;10(4):663‐673. 10.21037/jgo.2019.02.09. 31392047 PMC6657333

[23] Bilimoria KY, Stewart AK, Winchester DP, Ko CY. The National Cancer Data Base: a powerful initiative to improve cancer care in the United States. Ann Surg Oncol. 2008;15(3):683‐690. 10.1245/s10434-007-9747-3. 18183467 PMC2234447

[24] Bilimoria KY, Bentrem DJ, Ko CY, Stewart AK, Winchester DP, Talamonti MS. National failure to operate on early stage pancreatic cancer. Ann Surg. 2007;246(2):173‐180. 10.1097/SLA.0b013e3180691579. 17667493 PMC1933550

[25] Ansari D, Gustafsson A, Andersson R. Update on the management of pancreatic cancer: surgery is not enough. World J Gastroenterol. 2015;21(11):3157‐3165. 10.3748/wjg.v21.i11.3157. 25805920 PMC4363743

[26] Neoptolemos JP, Stocken DD, Friess H, et al. A randomized trial of chemoradiotherapy and chemotherapy after resection of pancreatic cancer. N Engl J Med. 2004;350(12):1200‐1210. 10.1056/NEJMoa032295. 15028824

[27] Neoptolemos JP, Stocken DD, Bassi C, et al. Adjuvant chemotherapy with fluorouracil plus folinic acid vs gemcitabine following pancreatic cancer resection: a randomized controlled trial. JAMA. 2010;304(10):1073‐1081. 10.1001/jama.2010.1275. 20823433

[28] Walston S, Salloum J, Grieco C, et al. Identifying clinical factors which predict for early failure patterns following resection for pancreatic adenocarcinoma in patients who received adjuvant chemotherapy without chemoradiation. Am J Clin Oncol. 2018;41(12):1185‐1192. 10.1097/COC.0000000000000452. 29727311 PMC6215749

[29] Raigani S, Ammori J, Kim J, Hardacre JM. Trends in the treatment of resectable pancreatic adenocarcinoma. J Gastrointest Surg. 2014;18(1):113‐123. 10.1007/s11605-013-2335-x. 24002769 PMC4137039

[30] Ostapoff KT, Gabriel E, Attwood K, Kuvshinoff BW, Nurkin SJ, Hochwald SN. Does adjuvant therapy improve overall survival for stage IA/B pancreatic adenocarcinoma? HPB. 2017;19(7):587‐594. 10.1016/j.hpb.2017.03.002. 28433254 PMC6324176

[31] Bergquist JR, Ivanics T, Shubert CR, et al. Type of resection (whipple vs. distal) does not affect the national failure to provide post‐resection adjuvant chemotherapy in localized pancreatic cancer. Ann Surg Oncol. 2017;24(6):1731‐1738. 10.1245/s10434-016-5762-6. 28070725

[32] Mittendorf EA, Vila J, Tucker SL, et al. The neo‐bioscore update for staging breast cancer treated with neoadjuvant chemotherapy incorporation of prognostic biologic factors into staging after treatment. JAMA Oncol. 2016;2(7):929. 10.1001/jamaoncol.2015.6478. 26986538 PMC5757376

[33] Bergquist JR, Murphy BL, Storlie CB, Habermann EB, Boughey JC. Incorporation of treatment response, tumor grade and receptor status improves staging quality in breast cancer patients treated with neoadjuvant chemotherapy. Ann Surg Oncol. 2017;24(12):3510‐3517. 10.1245/s10434-017-6010-4. 28828583

[34] Sohal D, McDonough S, Ahmad SA, et al. SWOG S1505: initial findings on eligibility and neoadjuvant chemotherapy experience with mfolfirinox versus gemcitabine/nab‐paclitaxel for resectable pancreatic adenocarcinoma. J Clin Oncol. 2019;37(4_suppl):414. 10.1200/jco.2019.37.4_suppl.414.

[35] Thadhani R. Formal trials versus observational studies. In: Mehta A, Beck M, Sunder‐Plassmann G eds. Fabry disease: perspectives from 5 years of FOS. Oxford: Oxford PharmaGenesis; 2006. https://www.ncbi.nlm.nih.gov/books/NBK11597/. 21290694

[36] Spigel DR. The value of observational cohort studies for cancer drugs. Biotechnol Healthc. 2010;7(2):18‐24. 22478817 PMC2899798

[37] Goulart BHL, Ramsey SD, Parvathaneni U. Observational study designs for comparative effectiveness research: an alternative approach to close evidence gaps in head‐and‐neck cancer. Int J Radiat Oncol Biol Phys. 2014;88(1):106‐114. 10.1016/j.ijrobp.2013.05.050. 24331656

[38] Visvanathan K, Levit LA, Raghavan D, et al. Untapped potential of observational research to inform clinical decision making: American Society of Clinical Oncology Research statement. J Clin Oncol. 2017;35(16):1845‐1854. 10.1200/JCO.2017.72.6414. 28358653

[39] Bergquist JR, Shah HN, Habermann EB, et al. Adjuvant systemic therapy after resection of node positive gallbladder cancer: time for a well‐designed trial? (results of a US‐national retrospective cohort study). Int J Surg. 2018;52:171‐179. 10.1016/j.ijsu.2018.02.052. 29496648

[40] Ghaneh P, Palmer DH, Cicconi S, et al. ESPAC‐5F: four‐arm, prospective, multicenter, international randomized phase II trial of immediate surgery compared with neoadjuvant gemcitabine plus capecitabine (GEMCAP) or FOLFIRINOX or chemoradiotherapy (CRT) in patients with borderline resectable pan. J Clin Oncol. 2020;38:4505. 10.1200/jco.2020.38.15_suppl.4505.

